# Greenery in the university environment: Students’ preferences and perceived restoration likelihood

**DOI:** 10.1371/journal.pone.0192429

**Published:** 2018-02-15

**Authors:** Nicole van den Bogerd, S. Coosje Dijkstra, Jacob C. Seidell, Jolanda Maas

**Affiliations:** 1 Department of Health Sciences, Faculty of Science, Vrije Universiteit Amsterdam, Amsterdam Public Health research institute, Amsterdam, the Netherlands; 2 Department of Neuro, Clinical & Developmental Psychology, Faculty of Behavioural and Movement Sciences, Vrije Universiteit Amsterdam, Amsterdam Public Health research institute, Amsterdam, the Netherlands; University of Sunderland, UNITED KINGDOM

## Abstract

A large body of evidence shows that interaction with greenery can be beneficial for human stress reduction, emotional states, and improved cognitive function. It can, therefore, be expected that university students might benefit from greenery in the university environment. Before investing in real-life interventions in a university environment, it is necessary to first explore students’ perceptions of greenery in the university environment. This study examined (1) preference for university indoor and outdoor spaces with and without greenery (2) perceived restoration likelihood of university outdoor spaces with and without greenery and (3) if preference and perceived restoration likelihood ratings were modified by demographic characteristics or connectedness to nature in Dutch university students (*N* = 722). Digital photographic stimuli represented four university spaces (lecture hall, classroom, study area, university outdoor space). For each of the three indoor spaces there were four or five stimuli conditions: (1) the standard design (2) the standard design with a colorful poster (3) the standard design with a nature poster (4) the standard design with a green wall (5) the standard design with a green wall plus interior plants. The university outdoor space included: (1) the standard design (2) the standard design with seating (3) the standard design with colorful artifacts (4) the standard design with green elements (5) the standard design with extensive greenery. Multi-level analyses showed that students gave higher preference ratings to the indoor spaces with a nature poster, a green wall, or a green wall plus interior plants than to the standard designs and the designs with the colorful posters. Students also rated preference and perceived restoration likelihood of the outdoor spaces that included greenery higher than those without. Preference and perceived restoration likelihood were not modified by demographic characteristics, but students with strong connectedness to nature rated preference and perceived restoration likelihood overall higher than students with weak connectedness to nature. The findings suggest that students would appreciate the integration of greenery in the university environment.

## 1. Introduction

People consistently value green environments more positively than environments without greenery [1–6]. Green outdoor environments are evaluated as more beautiful [3], and they are highly preferred over built outdoor environments [1]. People also appear to have more favorable attitudes toward a walk in a forest than toward a walk in a city center [6], and people who viewed local nature photographs were more satisfied with the conditions of their local environment than people viewing local built photographs [2]. Even the integration of greenery in an urban built environment can improve the preference for this environment. This is demonstrated by a recent study that showed that residential buildings with some type of integrated greenery are more preferred than residential buildings without integrated greenery [7]. Moreover, indoor spaces that contain greenery are perceived as more attractive than indoor spaces without [8–10]. Next to higher preference ratings, green environments are consistently perceived as more restorative than built environments [3, 6, 11–13]. In other words, green environments are perceived to be more beneficial for the recovery from stress and mental fatigue.

In agreement with the positive perceptions of green environments, there seems to be increased interest in the integration of greenery in built environments. In the Netherlands, various (research) projects stimulate the integration of greenery in cities [14], at elementary school playgrounds [15], and in hospitals [16]. It seems that this trend is not yet adopted by universities and that university students might benefit from greenery in the university environment as studying can be stressful [17, 18]. Before investing in real life interventions, it is necessary to first find out whether students would prefer a green university environment over a built university environment. This study, therefore, assessed students’ preferences with regard to greenery in the indoor and outdoor university environment. Because students often use the university outdoor environment for relaxation and stress reduction [19–21], this study also assessed the restoration likelihood of greenery in the university outdoor space. As the university student population is diverse, it was also assessed whether students’ preferences and perceived restoration likelihood were influenced by their demographic characteristics.

### 1.1. Preference and restoration likelihood

The literature on environmental preference and restoration is generally guided by Stress Recovery Theory from Ulrich (SRT) [22, 23] and Attention Restoration Theory (ART) from Kaplan and Kaplan [24, 25]. SRT is based on a psycho-evolutionary perspective, and suggests that interaction with an environment triggers and initiates an instant, unconsciously emotional response (affect). This emotional response influences functioning or behaviors that protect well-being and survival [22, 26]. For example, when seeing a bear during a walk in nature, the initial affect reaction (fear) can motivate avoidance. In many other situations, affective responses elicit adaptive functions that are not expressed in actions [26]. The positive affective response people experience from interaction with unthreatening greenery effects physical and psychological functioning related to relaxation and helps to block negative thoughts and moods [22, 26]. These affective responses can induce changes in physical and psychological states for stressed individuals, and keep emotional resources in an optimal state for unstressed individuals [22, 23]. According to SRT, positive affective responses to environments are more likely when an environment includes moderate to high complexity, structural properties that establish a focal point, moderate to high levels of depth, an even ground service, a curved line of site, and when the environment is perceived as safe, clear, and recognizable [22].

ART describes restoration as a process in which persons recover from mental fatigue. Directed attention enables persons to focus on tasks that require mental effort such as concentrating on difficult tasks while avoiding distractions. The capacity to direct attention may become fatigued with prolonged use, when there is little intrinsic motivation, and when suppressing distractions. When the directed attention capacity becomes fatigued, this may lead to errors, difficulty concentrating, irritability, and other symptoms of mental fatigue. According to ART, people can recover from this mental fatigue in green environments because nature engages attention in an effortless and involuntary manner. This involuntary attention allows the directed attention capacity to rest and restore. In addition to involuntary attention, nature contains certain components that evoke restorative experiences. First, nature provides a sense of being away from daily setting. Second, attending to several fascinating patterns in nature, such as the motion of the leaves, encourage involuntary attention (soft fascination). Third, nature allows for feelings of being in another world (extent). Fourth, compatibility between individuals needs and the functional aspects of nature [24, 25].

SRT [22] and ART [25] describe environmental preference as an immediate positive response (affect) that precedes and is closely related to restoration. This immediate response is based on peoples’ underlying needs. According to both theories, environments are evaluated by people in terms of its agreements with these underlying needs [22, 25]. Environments that offer functional qualities that are in agreement with the underlying needs are more likely to be preferred [22, 25]. Thus, preference does not only imply an attractive setting, it also includes instant pleasurable feelings and a neurophysiological reaction that can motivates avoidance or willingness to visit [26]. This present study defines preference as a setting that is attractive, pleasant, and a setting people are willing to visit [12, 27].

### 1.2. The need for greenery in the university environment

University students spend a lot of time in and around the university environment [28]. During their time at university, students are required to pay attention, take exams, or complete assignments. These tasks might call upon their directed attention resources, which accordingly could elicit mental fatigue or might raise their stress levels. It is, therefore, not surprising that the most reported stressors among students include their study and factors related to their study such as living up to expectations, financial issues, and lack of time [17, 18]. Additionally, stress and other psychological problems are reported among students [29, 30]. For example, in the United Kingdom [29] the percentage of self-reported psychological symptoms in students increased by 11% from admission at the start of the first academic year to the middle of the second academic year. In the second academic year, students reported significantly higher levels of anxiety and depression, and although these levels decreased in the third academic year they were still higher than in the first year [29]. In the Netherlands, 12% of the persons aged between 18–25 years [31] and 22% of the university students report psychological problems [32]. Stress and other psychological problems among students raise concerns as it could negatively impact their academic performances and present and later-life physical and psychological health [18, 33–35].

Students’ psychological health might benefit from a university environment that contains greenery. Several literature reviews [36–41] have already recognized the importance of greenery in the indoor and outdoor environment for stress reduction [37, 38, 40], changes in emotional states (e.g. more happiness or less anxiety) [36, 38, 41], and improved cognitive function [38, 40]. Only a few studies have investigated the beneficial effects of greenery in the university environment. A questionnaire study, conducted in the United Kingdom on a university campus with various green spaces, showed that the use of campus greenery positively correlates with students’ perceived quality of life [42]. Two other studies have shown that interior plants or window nature views in university classrooms positively influences students’ course and instructor evaluations [43, 44] and their academic results [43]. Although it can be expected that students would benefit from greenery in the university environment, it is unknown whether students prefer green university environments and perceive green university outdoor spaces to provide restorative benefits. Exploring students’ preferences for greenery in the university environment might, as suggested by the SRT and the ART, provide some insight into the need for greenery in the university environment. Additionally, exploring the restoration likelihood of outdoor university spaces may provide a first indication of the effectiveness of greenery in the university environment.

### 1.3. Actual and simulated greenery

The preference for green spaces over built spaces is well established [1–6]. Yet, there is also some evidence that simulated nature in indoor spaces can evoke more positive perceptions and feelings of restoration [9, 45]. A study conducted among university students compared the perceived restoration likelihood of indoor study-break spaces containing no views of nature, window views of nature with built elements present, and views of a nature poster. That study showed that students rated the study-break spaces that included nature posters as most restorative followed by study-break spaces with window views of nature [45]. Another study showed that patients perceived less stress and more attractiveness in hospital waiting rooms with interior plants or a nature poster compared to waiting rooms without green elements [9].

Next to actual green and nature images, multiple studies have recognized that colors can influence emotions and feelings [46, 47]. For example, the colors red, yellow, green, blue, and purple are associated with positive emotional responses among college students [48]. To our knowledge, little attention has been paid to the differences in perceptions between environmental designs with greenery and designs with colors. To gain insight into these differences, this present study included stimuli conditions with greenery, nature images, and stimuli with colors.

### 1.4. Demographic differences in preference and restoration likelihood

According to a literature review of Stamps that was published in 1999 [49], there is a high degree of consensus in environmental preference between many demographic subgroups. This literature review stated that there are little differences in environmental preference between demographic subgroups such as gender, ethnic groups, and political affiliation [49]. However, studies published after 1999 investigating preferences on gardens, wilderness, and other natural environments have shown differences by gender, age, income, education level, and profession [50–55].

Environmental preferences and perceived restoration likelihood ratings might also differ between individuals with different connectedness to nature. Two studies have revealed that individuals with a stronger connectedness to nature, nature hobbies, preferences for nature holidays, or positive childhood nature experiences were more likely to prefer natural environments [55], and more likely to report a higher perceived restoration likelihood of greenery [56]. Thus, there might be differences between certain subgroups in environmental preferences and perceived restoration likelihood. However, the literature is not consistent, and it is not clear if these differences are also present in university students. This study aimed to identify potential differences in preferences and perceived restoration likelihood between subgroups based on age, gender, education level, study discipline, and connectedness to nature.

### 1.5. The present study

In this study, digitally edited photographs were used to explore students’ perceptions of greenery in the university environment. The first objective was to investigate the difference in preference of Dutch university students for indoor and outdoor university spaces with and without greenery. The second objective was to investigate the difference in perceived restoration likelihood of Dutch university students on university outdoor spaces with and without greenery. The third objective was to investigate if preference and perceived restoration likelihood were modified by students’ age, gender, education level, study discipline, and connectedness to nature.

This study focused on university spaces where students are likely to spend most time, namely: a lecture hall, a classroom, a study area, and a university outdoor space. For each given space, the differences between standard designs typically used for those university spaces, a design with a colorful poster or colorful artifacts, and designs with greenery were assessed. The inclusion of the colorful designs allowed us to explore whether merely changing the standard design lead to differences in ratings and whether greenery was preferred over other changes to the standard design. For the indoor spaces (lecture hall, classroom, study area), the designs with greenery included a nature poster or actual greenery such as a green wall and interior plants.

## 2. Materials and method

This cross-sectional study is part of the Green Healthy Students Research. The aim of this research is to identify students’ needs with regard to greenery and fruit and vegetables in the study environment, and to examine the effects of green and fruit and vegetables interventions. The study protocol of this present study was approved by the Medical Ethical Committee of the VU Medical Centre in Amsterdam, and written consent was obtained from each participant.

### 2.1. Data collection and participants

Data were collected by means of an online and identical paper questionnaire between February and March 2016. There was a Dutch version and an English version available. The communications departments of all 13 public universities in the Netherlands were contacted by the researchers to ask if they were willing to distribute the questionnaire among their students by placing a recruitment text, with internet hyperlink to the questionnaire, on their student information webpage. The recruitment text invited students to give their opinion on their ideal university environment. The recruitment text did not specify that there was a special interest in greenery. In total, eight universities placed the recruitment text with internet hyperlink on their student information webpage (Eindhoven University of Technology, Erasmus University Rotterdam, Leiden University, Radboud University Nijmegen, University of Amsterdam, University of Twente, Vrije Universiteit Amsterdam, Wageningen UR). These universities vary in academic fields and in locations (inner city versus outskirts). Student unions of these eight universities were also asked to spread the recruitment text via their social media. The online questionnaire could be accessed on all devices with internet. One of the researchers (NvdB) and a number of students visited six out of eight universities once where they distributed the paper questionnaire at the university canteens and restaurants. None of these canteens or restaurants contained extensive green elements.

In total 1,069 students accessed the questionnaire and the completion value was 70% (*N* = 749). Students who did not complete the whole questionnaire were excluded from this study. In the Netherlands, universities focus on academic and research-oriented education. Students who did not follow an academic and research oriented course were excluded (i.e. PhD and college students) (*N* = 4). An additional 23 students were excluded because they were not enrolled at one of the eight included universities. After exclusion, 722 participants were included in the statistical analyses.

Of the 722 students that were included in this study, 206 (28.5%) studied at Vrije Universiteit Amsterdam, 149 (20.6%) studied at Erasmus University Rotterdam, 132 (18.3%) studied at Eindhoven University of Technology, 79 (10.9%) studied at Leiden University, 65 (9.0%) studied at the University of Amsterdam, 55 (7.6%) studied at the University of Twente, 24 (3.3%) studied at Radboud University Nijmegen, and 12 (1.7%) studied at Wageningen UR. About half of the sample (*N* = 372, 51.5%) filled out the online version questionnaire. Table 1 shows the demographic characteristics. The sample comprised 261 males and 460 females, and the age ranged from 18 to 65 year with a median of 21 years.

**Table 1:** Characteristics of the study population (N = 722).

	All universities pooled	
**Age (Median; IQR)**	21	(20–23)
**Gender—Male (N; %)**	261	(36.1)
**Ethnicity–Dutch (N; %)**	519	(71.9)
**Current education level (N; %)**		
Bachelor	446	(61.8)
Master	243	(33.7)
Premaster / transition year	33	(4.6)
**Study discipline (N; %)**		
Health-related studies	152	(21.1)
Humanities and social science studies	231	(32.0)
Economics and Law studies	195	(27.0)
Technical studies	138	(19.1)
**Connectedness to nature–low (N; %)**	377	(52.2)

### 2.2. Stimuli

**Fig 1:**
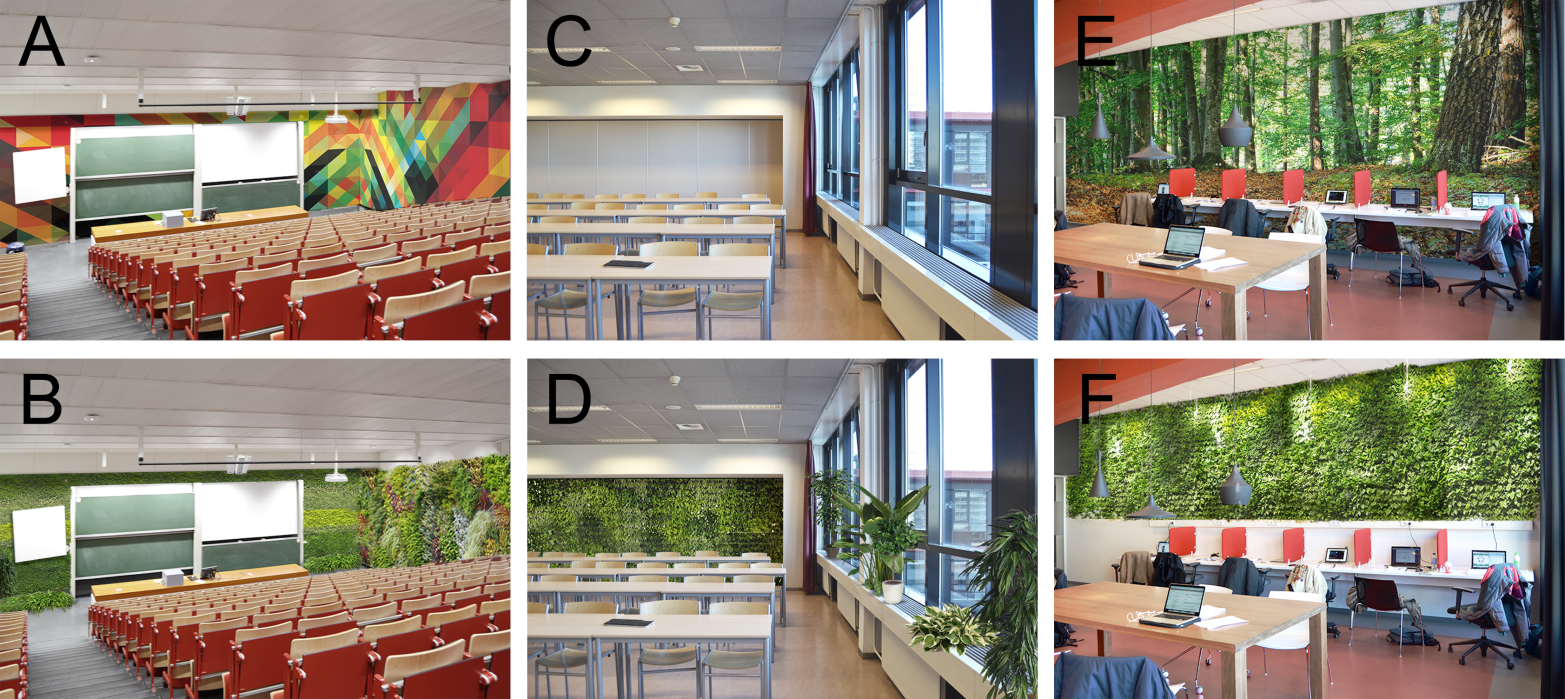
Examples of the indoor space designs. (A) Lecture hall with colorful poster; (B) Lecture hall with green wall; (C) Classroom with standard design; (D) Classroom with green wall plus interior plants; (E) Study area with nature poster; (F) Study area with green wall. *Reprinted from Burton Hamfelt Architects under a CC BY license*, *with permission from Burton Hamfelt*, *original copyright 2016*.

Students’ preference and restoration likelihood were assessed with photographs that were integrated into the questionnaire. The environmental stimuli included three indoor spaces and one university outdoor space. The three indoor spaces were a lecture hall, a classroom, and a study area. For each indoor university space five (or four for the lecture hall) different designs were created (Fig 1). The first photograph depicted the standard design of the three indoor spaces at the Vrije Universiteit Amsterdam. This photograph was used as a reference category. The other photographs were identical to the photograph with the standard design, but they were digitally edited. In the second photograph, the back wall or the side wall of the indoor space was replaced with a colorful image or a colorful urban scene image reflecting a wall sized colorful poster. In the third photograph, the back wall or the side wall of the indoor space was replaced with an image of a natural scene reflecting a wall sized nature poster. In the fourth photograph, the back wall or the side-wall of the indoor space was replaced with a partly or wall sized green wall. In the fifth photograph, interior plants were edited into the photograph with the green wall. For the lecture hall, no design with a green wall plus interior plants was created because the lecture hall photograph had not enough open space to place additional interior plants.

**Fig 2:**
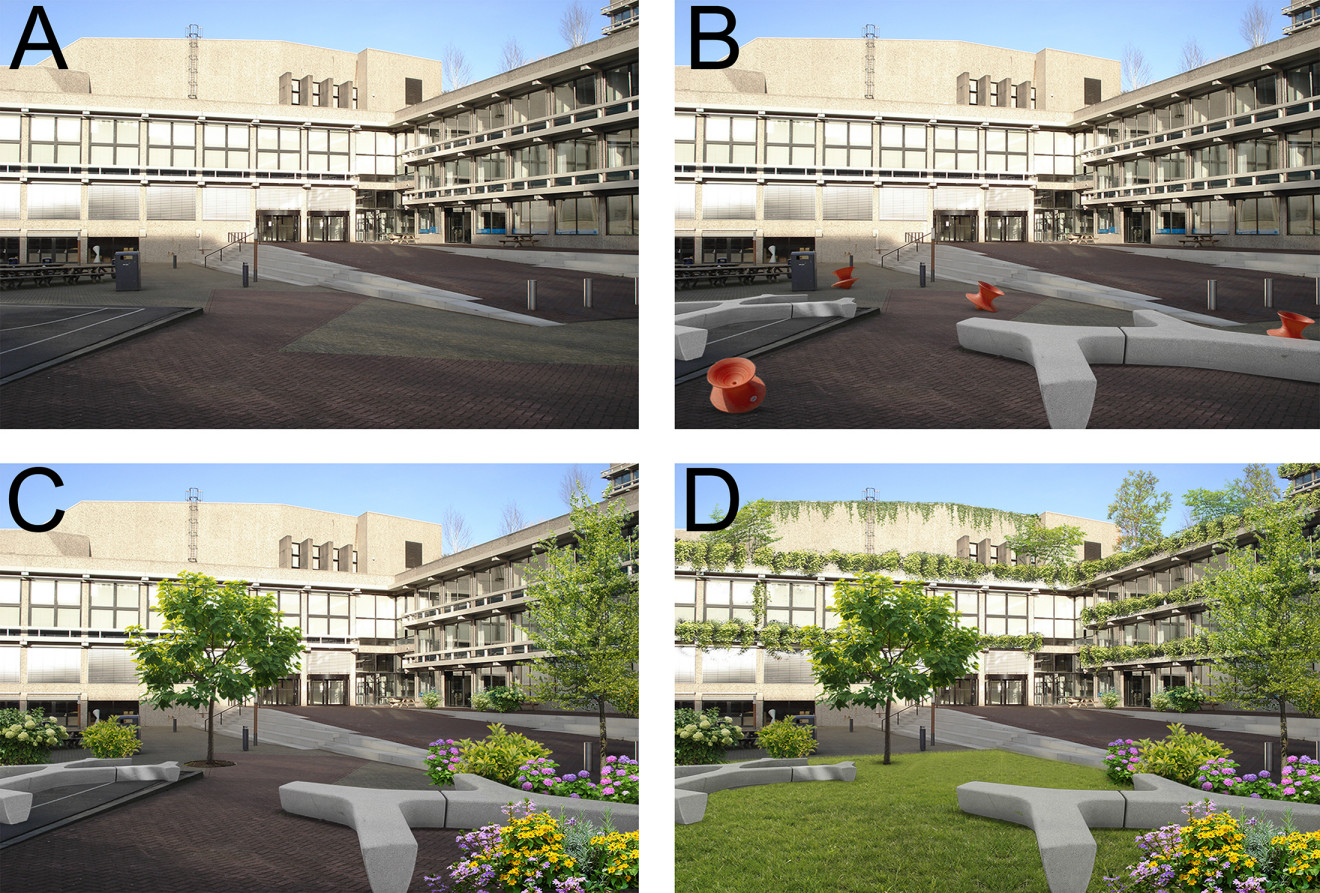
University outdoor space designs. (A) Standard design; (B) Design with built seating and colorful artifacts; (C) Design with built seating and green elements; (D) Design with built seating and extensive greenery. *Reprinted from Burton Hamfelt Architects under a CC BY license*, *with permission from Burton Hamfelt*, *original copyright 2016*.

For the university outdoor space four designs were created (Fig 2). The first photograph depicted the standard design of an outdoor space at the Vrije Universiteit Amsterdam. This outdoor space is used for study breaks, relaxation, and social interaction. It includes only built elements such as picnic tables, streetlights, and trash bins. This photograph was used as a reference category. The other photographs were identical to the photograph with the standard design, but they were digitally edited. In the second photograph, built seating and built colorful artifacts were edited into the photograph with the standard design. In the third photograph, built seating and green elements were edited into the photograph with the standard design. Green elements included flowerbeds, plants, and trees. In the fourth photograph, built seating and extensive greenery were edited into the photograph with the standard design. The extensive greenery included flowerbeds, plants, trees, building integrated greenery, and a small lawn.

### 2.3. Materials

The questionnaire consisted of multiple sections. Next to questions about demographic background and the photograph judgements, the questionnaire also included questions on lifestyle and the current university environment. The questions on lifestyle and the current university environment where outside the scope of this study, and will not be discussed. Completion of the questionnaire took approximately 10–15 minutes, and it was briefly pilot tested by 10 Master and Bachelor students.

#### 2.3.1. Preference

Preference was measured using three items that have been used in previous studies [12, 27], and described two aesthetic properties and one behavioral property. The items were as following: “The setting is pleasant” [12]; “The setting is attractive” [12, 27]; ‘I would like to: be educated in / study in / visit this setting” [27]. For the third item, “like to be educated in” was used for the lecture hall and classroom, “like to study in” was used for the study area, and “like to visit” was used for the university outdoor space. Students rated the items on a five point Likert scale ranging from 0 (strongly disagree) to 4 (strongly agree). Reliability was tested with Cronbach’s alpha, and showed adequate reliability varying between α = 0.88 and α = 0.96 for the four spaces [57]. Average scores were created ranging from zero to four with a higher score reflecting stronger preference.

#### 2.3.2. Perceived restoration likelihood

Perceived restoration likelihood was only measured for the university outdoor space, and was measured using three items that were adapted from previous studies to match research purposes [12, 58]. The first item assessed likelihood of restoration given the condition of attentional fatigue [12]: “If, at the end of a week of exams and intense study, I am mentally exhausted and unable to concentrate, than I would regain my concentration in this setting”. The second and third item reflected perceived recovery when staying in the setting for 20 minutes [12, 58]. These two items were: “If I would stay in this setting for 20 minutes I would feel that (1) I had come to rest (2) that I have renewed energy”. Students rated the three items on a five-point scale ranging from 0 (strongly disagree) to 4 (strongly agree). Cronbach’s alpha showed adequate reliability (α = 0.91) [57]. An average score was created ranging from zero to four with a higher score reflecting stronger perceived restoration likelihood.

#### 2.3.3. Potential effect modifiers

There were five demographic variables identified as potential effect modifiers: gender (male, female), age in years, ethnicity (Dutch, non Dutch [59]), current education level, and study discipline. These variables were assessed with items from the Dutch Student Monitor Questionnaire [60]. Current education level was categorized according to the Dutch academic system: Bachelor, Master, Pre-master/transition year. There was an answer possibility “other”, but those students were excluded from this study. Study discipline was initially assessed with an open-ended question, and thereafter categorized in: health-related studies; humanities and social science studies; economics and law studies; and technical studies.

Another potential effect modifier was the self-reported connectedness to nature. Although multiple connectedness to nature scales exist, we used a slightly adjusted version of the single item scale CN-SI [61]: “To what extend are you a nature lover”. Students rated this item on a ten-point scale ranging from 1 (very low) to 10 (very high). We used a single item because connectedness to nature was not a primary outcome of this study, and we wanted to ensure a reasonable questionnaire length. Connectedness to nature was identified as an effect modifier and, therefore, stratified with a mean split (mean = 7.22, SD = 1.72). Scores lower than eight indicated weak connectedness to nature; scores of eight or higher indicated strong connectedness to nature.

### 2.4. Procedure

In the online questionnaire, students were randomly assigned to one design per university space. For example, a student could have seen the lecture hall with the standard design, the classroom with the green wall, the study area with the colorful poster, and the outdoor space with built seating and extensive greenery. For the paper version questionnaire we created various versions in both English and Dutch. Each version showed all university spaces, but the designs varied in each version. We ensured that in each version no double designs were presented; for example, students never rated the standard design of two university spaces. The number of students per condition is presented in the results section.

Prior to rating the designs on preference and restoration likelihood, written instructions were provided on how to rate the designs. Students were instructed to judge the space depicted on the photograph and not the quality of the photograph itself [62]. Students first viewed the photograph and accompanying questions could be found underneath. Each photograph and accompanying questions were placed on a separate (web) page. The image size of the photograph was 900x600 pixels, and the actual size of the photographs in the paper version questionnaire was 10 by 15 centimeters. In the online version, the photograph size differed depending on the type of device used (e.g. mobile phone or laptop). The photographs were taken and digitally edited by an architect who is specialized in urban architecture and greenery designs for the purpose of this study.

### 2.5. Statistical analyses

Linear multi-level regression analyses were used to analyze the associations between the various designs per university space and the outcome variables preference and perceived restoration likelihood. Each university space was assessed by a separate association model; the predictor variables were the various designs belonging to the given university spaces. A two level structure was used; the first level corresponded to the individual students and the second level corresponded to the universities. Multi-level analyses were used to take possible clustering of students observations within universities into account [63]. We expected clustering because the universities varied in inner city and outskirt locations and academic fields. The necessity of random intercepts and random slopes were assessed by a likelihood ratio test [63]. All association models included a random intercept; none of the models included random slopes. The intraclass correlation coefficient (ICC) was estimated by dividing the variance between the universities by the total variance, where the total variance was defined as the overall error variance and the variance between the universities. This estimated ICC provides an indication of how much of the total variance in preference or restoration likelihood is accounted by the clustering within universities [63].

Effect sizes are expressed as regression coefficients (β) with their 95% confidence intervals (95% CI). Interaction terms were used to examine if gender, age, ethnicity, current education level, study discipline, and connectedness to nature were effect modifiers. A variable was considered an effect modifier when the interaction term was below the p-value threshold of 0.05.

Secondary analyses were performed to analyze if there were differences in preference and restoration likelihood between designs that included greenery and the designs with the colorful poster or built colorful artifacts. In these analyses, the designs with the colorful poster or with the built colorful artifacts were set as the reference category. Data preparations and reliability analyses were performed with SPSS 23. Multi-level regression analyses were performed with MLwiN 2.31. A p-value lower than 0.05 was considered to indicate statistical significance.

## 3. Results

Multi-level linear analyses with random intercepts were used to account for possible dependencies between observations within a university. Although only the study area model showed a statistically significant (p-value <0.05) likelihood ratio test for the university level intercept, we continued with multi-level analyses with a random intercept for the university level in all association models. The magnitude of clustered observations within universities is reflected by the ICC. The regression coefficients (β) represent the difference in preference or restoration likelihood compared to the reference category.

### 3.1. Preference for three university indoor spaces

Table 2 presents descriptive statistics and results of the linear multi-level regression analyses for the lecture hall. The lecture hall with the nature poster received the highest preference ratings with 0.88 (95% CI = 0.66–1.10) points higher than the lecture hall with the standard design. The lecture hall with the nature poster was followed by the lecture hall with the green wall (β = 0.49, 95% CI = 0.27–0.71) and the lecture hall with the colorful poster (β = 0.23, 95% CI = 0.01–0.44).

**Table 2:** Multi-level associations between students’ preference ratings and various designs of a lecture hall.

	N	Mean (SD)	β	95% CI
**Standard design**	167	1.53 (0.76)	ref	ref
**Colorful poster**	166	1.76 (1.05)	0.23	0.01–0.44*
**Nature poster**	155	2.42 (1.02)	0.88	0.66–1.10*
**Green wall**	166	2.02 (1.19)	0.49	0.27–0.71*
**Overall**	654	1.92 (1.07)		

* p-value regression coefficient <0.05

ref = reference category, estimated ICC = 0.01, preference was rated on a scale 0 (strongly disagree) to 4 (strongly agree).

Table 3 presents descriptive statistics and results of the linear multi-level regression analyses for the classroom. The classroom association model showed a similar trend as the lecture hall association model. The classroom with the nature poster received the highest preference ratings with 0.76 (95% CI = 0.58–0.93) points higher than the classroom with the standard design. The classroom with the colorful poster received the lowest preference ratings (β = 0.44, 95% CI = 0.27–0.62).

**Table 3:** Multi-level associations between students’ preference ratings and various designs of a classroom.

	N	Mean (SD)	β	95% CI
**Standard design**	143	2.19 (0.83)	ref	ref
**Colorful poster**	146	2.63 (0.77)	0.44	0.27–0.62*
**Nature poster**	146	2.95 (060)	0.76	0.58–0.93*
**Green wall**	136	2.95 (0.74)	0.76	0.59–0.94*
**Green wall + interior plants**	150	2.94 (0.83)	0.75	0.58–0.93*
**Overall**	721	2.73 (0.81)		

* p-value regression coefficient <0.05

ref = reference category, estimated ICC = 0.01, preference was rated on a scale 0 (strongly disagree) to 4 (strongly agree).

Table 4 presents descriptive statistics and results of the linear multi-level regression analyses for the study area. The study area with the green wall received the highest preference ratings with 0.90 (95% CI = 0.41–0.79) points higher than the study area with the standard design. The study area with the green wall was followed by the study area with the nature poster (β = 0.65, 95% CI = 0.45–0.85) and the study area with the green wall plus interior plants (β = 0.53, 95%CI = 0.32–0.73).

**Table 4:** Multi-level associations between students’ preference ratings and various designs of a study area.

	N	Mean (SD)	β	95% CI
**Standard design**	142	2.27 (0.93)	ref	ref
**Colorful poster**	141	2.60 (0.88)	0.33	0.13–0.53*
**Nature poster**	132	2.92 (0.79)	0.65	0.45–0.85*
**Green wall**	168	2.88 (0.81)	0.60	0.41–0.78*
**Green wall + interior plants**	137	2.82 (0.94)	0.53	0.32–0.73*
**Overall**	720	2.70 (0.90)		

* p-value regression coefficient <0.05

ref = reference category, estimated ICC = 0.02, preference was rated on a scale 0 (strongly disagree) to 4 (strongly agree).

Secondary analyses showed that preference of all indoor designs with the nature poster, green wall, and the green wall plus interior plants were statistically significant higher than the preference ratings of the designs with the colorful poster of those given spaces (S1 Tables). There was one exception: the study area with the green wall plus interior plants was not rated statistically significant higher than the study area with the colorful poster (β = 0.20, 95% CI = -0.004–0.40).

### 3.2. Preference and perceived restoration likelihood of the university outdoor environment

Table 5 presents descriptive statistics and results of the linear multi-level regression analyses for the university outdoor space. The university outdoor space with built seating and extensive greenery received the highest preference ratings with 2.09 (95% CI = 1.92–2.25) points higher than the standard design. The university outdoor space with built seating and extensive greenery was followed by the design with the built seating and green elements (β = 1.76, 95% CI = 1.59–1.93) and the design with the built seating and colorful artifacts (β = 0.51, 95% CI = 0.35–0.68). The associations with perceived restoration likelihood of the university outdoor space followed a similar trend. The university outdoor space with the built seating and extensive greenery received the highest perceived restoration likelihood rating with 1.58 (95% CI = 1.40–1.76) points higher than the standard design.

**Table 5:** Multi-level associations between students’ preference and perceived restoration likelihood ratings and various designs of a university outdoor space.

	N	Preference			Restoration likelihood		
		Mean (SD)	β	95% CI	Mean (SD)	β	95% CI
**Standard design**	165	1.28 (0.86)	ref	ref	1.20 (0.81)	ref	ref
**Built seating and colorful artifacts**	175	1.79 (0.90)	0.51	0.35–0.68*	1.46 (0.84)	0.26	0.08–0.43*
**Built seating and green elements**	143	3.04 (0.66)	1.76	1.59–1.93*	2.34 (0.84)	1.14	0.95–1.33*
**Built seating and extensive greenery**	171	3.36 (0.63)	2.09	1.92–2.25*	2.78 (085)	1.58	1.40–1.76*
**Overall**	654	2.35 (1.16)			1.93 (1.05)		

* p-value regression coefficient <0.05

ref = reference category, estimated ICC preference = 0.01, estimated ICC perceived restoration likelihood = 0.01, preference and restoration likelihood were rated on a scale 0 (strongly disagree) to 4 (strongly agree).

Secondary analyses showed that preference and perceived restoration likelihood of the university outdoor space with the green elements and extensive greenery were statistically significant higher than the ratings of the design with the built seating and colorful artifacts (S1 Table).

### 3.3. Modifying variables

Interaction terms showed that there was no effect modification by gender, age, ethnicity, current education level, or study discipline (p-value interaction term > 0.05). Yet, effect modifications for the self-reported connectedness to nature (p-value interaction term <0.05) were found. Stratified by subgroups of weak and strong connectedness to nature, the association models of the three indoor spaces followed a similar trend as the un-stratified analyses (see Table 6). In both subgroups, preference for the indoor spaces with the colorful poster, nature poster, green wall, and green wall plus interior plants was statistically significant higher than those of the given spaces with the standard design. There was one exception: in the subgroup with weak connectedness to nature the lecture hall with the colorful poster was not rated higher on preference than the standard design. Students with a strong connectedness to nature gave overall higher preference ratings to the indoor spaces than those with a weak connectedness to nature.

**Table 6:** Multi-level associations between students’ preference ratings and various designs of a lecture hall, classroom, and a study area analyzed by subgroups of connectedness to nature.

	Lecture hall			Classroom			Study area		
	*N*	β	95% CI	*N*	β	95% CI	*N*	β	95% CI
**Standard design**									
Weak connectedness to nature	123	ref	ref	79	ref	ref	68	ref	ref
Strong connectedness to nature	111	ref	ref	62	ref	ref	73	ref	ref
**Colorful poster**									
Weak connectedness to nature	86	0.10	-0.28–0.30	72	0.56	0.31–0.80*	76	0.32	0.05–0.60*
Strong connectedness to nature	78	0.36	0.04–0.67*	73	0.33	0.08–0.57*	64	0.29	0.004–0.57*
**Nature poster**									
Weak connectedness to nature	78	0.54	0.25–0.84*^±^	68	0.70	0.45–0.94*	68	0.36	0.08–0.64*^±^
Strong connectedness to nature	77	1.20	0.89–1.51*	75	0.80	0.56–1.05*	64	0.93	0.65–1.21*
**Green wall**									
Weak connectedness to nature	90	0.14	-0.15–0.43	70	0.64	0.40–0.89*^±^	89	0.41	0.14–0.67*
Strong connectedness to nature	71	0.86	0.54–1.18*	67	0.89	0.64–1.14*	78	0.81	0.54–1.08*
**Green wall + interior plants**									
Weak connectedness to nature				88	0.70	0.46–0.93*	75	0.37	0.09–0.64*^±^
Strong connectedness to nature				60	0.90	0.64–1.15*	58	0.73	0.43–1.02*

* p-value regression coefficient <0.05

^±^ p-value interaction term <0.05

ref = reference category, preference was rated on a scale 0 (strongly disagree) to 4 (strongly agree).

Stratified by subgroups of weak and strong connectedness to nature, the association models for preference and perceived restoration likelihood in the university outdoor environment followed a similar trend as the un-stratified analyses (see Table 7). In both subgroups, preference and perceived restoration likelihood were statistically significant higher for the university outdoor space with the colorful artifacts and the university outdoor spaces with greenery than the preference ratings of the standard design. There was one exception: in the subgroup with weak connectedness to nature the perceived restoration likelihood of the university outdoor space with the built seating and colorful artifacts was not rated statistically significant higher than the standard design. Students with a strong connectedness to nature gave overall higher preference and perceived restoration likelihood ratings than those with a weak connectedness to nature.

**Table 7:** Multi-level associations between students’ preference and (perceived restoration likelihood ratings and various designs of a university outdoor space analyzed by subgroups of connectedness to nature.

	Preference			Restoration likelihood	
	*N*	β	95% CI	β	95% CI
**Standard design**					
Weak connectedness to nature	77	ref	ref	ref	ref
Strong connectedness to nature	87	ref	ref	ref	ref
**Built seating and colorful artifacts**					
Weak connectedness to nature	95	0.38	0.15–0.61*	0.19	-0.05–0.43
Strong connectedness to nature	79	0.61	0.38–0.84*	0.30	0.04–0.56*
**Built seating and green elements**					
Weak connectedness to nature	80	1.73	1.49–1.97*	1.12	0.87–1.37*
Strong connectedness to nature	61	1.74	1.49–1.99*	1.14	0.86–1.42*
**Built seating and extensive greenery**					
Weak connectedness to nature	83	1.84	1.60–2.07*^±^	1.41	1.17–1.66*^±^
Strong connectedness to nature	86	2.32	2.10–2.55*	1.74	1.48–1.99*

* p-value regression coefficient <0.05

^±^ p-value interaction term <0.05

ref = reference category, preference and restoration likelihood were rated on a scale 0 (strongly disagree) to 4 (strongly agree).

## 4. Discussion

### 4.1. Main findings

The objectives of this study were to investigate if preference and perceived restoration likelihood of Dutch university students differed between university spaces with and without greenery, and to explore if these outcomes differed between subgroups. This study showed that university students gave higher preference to university environments that included some type of greenery than to university environments without greenery. University students also gave higher perceived restoration likelihood ratings to university outdoor spaces that included greenery than those without. Preference ratings and the perceived restoration likelihood ratings were modified by connectedness to nature.

University students preferred indoor spaces with some type of greenery (i.e. nature poster, green wall or green wall plus interior plants) over indoor spaces without greenery (i.e. the standard design or with a colorful poster). Students also gave higher preference ratings to the outdoor spaces with some green elements and extensive greenery than to the outdoor spaces with the standard design or with built seating and colorful artifacts. This aids to the results of other studies that consistently have shown that samples of European, North American, and Asian adults prefer green environments over environments without greenery [1–5, 22]. Yet, those studies have mainly focused on exploring the differences between completely natural environments (e.g. forests, parks) and built environments. The present study differs from those studies as it investigated if greenery in different university spaces was a valued addition.

The overall preference ratings of the lecture hall designs were lower than the preference ratings of the various designs of the classroom, study area, and university outdoor space. Only the lecture hall with the nature poster was rated higher than “neutral” on the preference scale. The reason for this is not clear, but it may be that students have a general dislike for lecture halls compared to classrooms, study areas, and university outdoor spaces.

Preference ratings for the indoor spaces with the nature poster were relatively high. For example, the lecture hall with the nature poster was rated higher on preference than the other designs of the lecture hall. Although there are differences in the design and measured outcome, a photograph study by Felsten et al [45] showed comparable results. Felsten et al [45] showed that study-break spaces with a nature poster were perceived higher in restoration likelihood than study-break spaces without greenery or mundane window nature views [45]. An explanation for the relatively high preference ratings of the indoor spaces with the nature poster might be found in the SRT described by Ulrich [22]. This framework asserts that preference is likely to be higher when natural environments contain certain components, including: structural aspects that establish a focal point, depth and openness, vegetation, environmental content [22, 26]. When critically reviewing the photographs used in this study, one can argue that the photographs of the indoor spaces with the nature poster contained more depth, structural aspects, and more variety in vegetation than the photographs of the indoor spaces with the green wall.

University students perceived the restoration likelihood of the university outdoor spaces that included greenery higher than the standard design and the design with the built seating and colorful artifacts. These results support the existing research on the restorative potential of greenery. Multiple studies have shown that green natural environments are perceived to be more restorative than built environments [3, 6, 11–13, 24]. The present findings are also consisted with findings from a recent study among Australian primary schoolchildren, which gave higher ratings of restoration likelihood to school playgrounds that contained a higher amount of greenery [64].

The preference and perceived restoration likelihood ratings were not modified by students’ gender, age, ethnicity, current education level, or study discipline. These results are in line with the literature review of Stamps [49], but contradict other studies that did find differences between demographic subgroups in preference or restoration likelihood [50–55]. In this present study, preference and perceived restoration likelihood were modified by self-reported connectedness to nature. It seemed that all student were more positive about university spaces that included some type of greenery; however, the subgroup with strong connectedness to nature showed overall higher preference and perceived restoration likelihood ratings than the subgroup with weak connectedness to nature. These results suggest that students with a strong connectedness to nature appreciate a green university more than students with weak connectedness to nature. Several previous studies have suggested that connectedness to nature can modify the associations between environmental stimuli and preferences or restoration likelihood [55, 56]. Additionally, there is some evidence that connectedness to nature mediates the association between exposure to greenery and well-being [65].

### 4.2. Strengths and limitations

This study was one of the first that examined students’ preference and perceived restoration likelihood of greenery in the university environment. The findings of this study are based on a large sample of university students of eight different universities in the Netherlands. The used measurements were not validated in a university student sample; nevertheless, they showed excellent internal consistency. Although the estimated ICC for each association model was very low [63], the likelihood ratio test showed that in the study area model the university level intercept had a significant influence on the model. This indicates that students are nested within universities, which is also demonstrated by the small deviation between the crude mean differences and the beta’s reflecting the real mean differences. Even though this deviation is small and not always present and considering that the included universities varied in academic fields and inner city versus outskirts locations, not controlling for the universities through multi-level analysis would have led to (minor) overestimations Overall, all students seem to prefer green university spaces over university spaces without greenery, yet these preference ratings might differ between students from different universities.

Experiences based on photographs could differ from the experiences in the real life settings [66]. Despite this concern, the use of photographs and digital manipulations provide a practical possibility to compare multiple stimuli conditions with experimental control. Therefore, the use of photographs and digital manipulations in this study were believed to be appropriate to achieve the research objectives. However, the use of this design might have allowed for demand characteristics [67]. If students firstly viewed three photograph conditions with greenery and thereafter a photograph condition with a standard built design, they might have formed an understanding of the study objectives and subconsciously altered their opinions to fit this understanding. To counter demand characteristics, this study used a between subjects design in which the students were randomly assigned to one of the photograph conditions per university space. Due to the chosen design and the large sample in this study, the potential effects of demand characteristics were believed to be minimal.

At all eight universities similar collecting methods were used, and data was collected via internet and paper versions questionnaires; nevertheless, the response rate at some universities was low. This might have resulted in selection bias, which accordingly may have lowered the representativeness of the results. Moreover, connectedness to nature was because of questionnaire length concerns measured with a single item scale that was not validated by previous studies. Connectedness to nature was dichotomized with scores lower than eight indicating low connectedness to nature. It should be noted that scores between six and eight on a ten point scale might be considered moderate. In this study scores lower than eight were already considered weak connectedness to nature based on the relatively high self-reported connectedness to nature ratings. As a consequence, it cannot be stated that the measurement used in this study provides an accurate representation of connectedness to nature, and thus the results of this measure might have been subject to measurement bias.

### 4.3. Implications and recommendations

The findings of this study add to the rationale for the implementation of greenery in the university environment. The results convincingly showed that actual greenery and nature posters in the university environment are preferred and perceived as more restorative by students than the standard design or a design with colors. These findings might convince, stimulate, or guide policy makers to integrate more greenery in the university environment. Additionally, the SRT [22] and ART [25] suggest that preference is an immediate response based on peoples’ underlying needs, and environments are evaluated in terms of its agreements with these underlying needs. The findings might, therefore, indicate the compatibility between the designs with greenery and the underlying needs of students. The SRT [22] and ART [25] also suggest that preference precedes and is closely related to restoration. Based on this, and strengthened by the existing literature on the restorative effects of greenery [36–41], the findings may provide an indication of the effectiveness of greenery in the university environment on students’ restorative experiences. However, replication of this study in a real-life setting is needed to investigate the effects of greenery in the university environment on students’ restorative experiences and psychological well-being.

The various greenery stimuli conditions were not rated equally; the stimuli conditions with the nature poster received the highest preference ratings. However, in a real life setting a nature posters might lack some properties that are considered to contribute to the beneficial effects of greenery including local climate and sensory aspects [40]. Future experimental research should compare various greenery designs to establish a clear understanding of which real-life conditions are most preferred. We further recommend that future studies also focus on the difference between interior plants and a green wall. Such understandings can further guide policy makers and greenery suppliers in creating optimal green university environments.

### 4.3. Conclusion

Taken together, greenery seems to be an appreciated addition to university environment by students. This study showed that students prefer university spaces with actual greenery or nature posters, and that they also expect that a green outdoor university environment can be more restorative. Further experimental research is needed to find out if the implementation of greenery in the university environment affects students’ psychological well-being.

## Supporting information

S1 Tables (A) Multi-level associations between students’ preference ratings on a scale from zero to four and various greenery designs of a lecture hall, classroom, and a study area compared to the design with the colorful poster (B) Multi-level associations between students’ (I) preference and (II) perceived restoration likelihood ratings on a scale from zero to four and various greenery designs of a university outdoor space compared to the design with built seating and colorful artifacts.
(DOCX)

S1 File Questionnaire in Dutch and English. (PDF)

S1 Dataset Dataset. (SAV)
