# Supplementary material for: Greenery in the university environment: Students’ preferences and perceived restoration likelihood
Source: PLoS One. 2018 Feb 15;13(2):e0192429. doi: 10.1371/journal.pone.0192429 (PMC5813944; doi:10.1371/journal.pone.0192429)
Supplement: S1 Tables — (A) Multi-level associations between students’ preference ratings on a scale from zero to four and various greenery designs of a lecture hall, classroom, and a study area compared to the design with the colorful poster (B) Multi-level associations between students’ (I) preference and (II) perceived restoration likelihood ratings on a scale from zero to four and various greenery designs of a university outdoor space compared to the design with built seating and colorful artifacts. (DOCX) [file pone.0192429.s001.docx]

# S1 Tables

**(A) Multi-level associations between students’ preference ratings and various greenery designs of a lecture hall, classroom, and a study area compared to the design with the colorful poster**

|  | Lecture hall | | | Classroom | | | Study area | | |
| --- | --- | --- | --- | --- | --- | --- | --- | --- | --- |
|  | N | β | 95% CI | N | β | 95% CI | N | β | 95% CI |
| **Colorful poster** | 166 | ref | ref | 146 | ref | ref | 142 | ref | ref |
| **Nature poster** | 155 | 0.66 | 0.44 – 0.88 | 146 | 0.32 | 0.15 – 0.49 | 132 | 0.32 | 0.12 – 0.53 |
| **Green wall** | 166 | 0.26 | 0.04 – 0.48 | 137 | 0.32 | 0.15 – 0.50 | 168 | 0.27 | 0.08 – 0.46 |
| **Green wall + interior plants** |  |  |  | 150 | 0.31 | 0.14 – 0.49 | 137 | 0.20 | -0.004 – 0.40 |

* *P-value regression coefficient* <0.05*,* ref= reference category, preference was rated on a scale 0 (strongly disagree) to 4 (strongly agree)*.*

**(B) Multi-level associations between students’ preference and perceived restoration likelihood ratings and various greenery designs of a university outdoor space compared to the design with built seating pieces and colorful artifacts**

|  | **Preference** | | | **Restoration likelihood** | |
| --- | --- | --- | --- | --- | --- |
|  | *N* | β | 95%CI | β | 95%CI |
| **Built seating pieces and colorful artifacts** | 175 | ref | ref | ref | ref |
| **Built seating pieces and green elements** | 143 | 1.25 | 1.08 – 1.42 | 0.88 | 0.70 – 1.07 |
| **Built seating pieces and extensive greenery** | 172 | 1.58 | 1.49 – 1.74 | 1.33 | 1.15 – 1.41 |

* *P-value regression coefficient* <0.05*,* ref= reference category, preference and restoration likelihood were rated on a scale 0 (strongly disagree) to 4 (strongly agree).
