# Supplementary material for: Greenery in the university environment: Students’ preferences and perceived restoration likelihood
Source: PLoS One. 2018 Feb 15;13(2):e0192429. doi: 10.1371/journal.pone.0192429 (PMC5813944; doi:10.1371/journal.pone.0192429)
Supplement: S1 File — (PDF) [file pone.0192429.s002.pdf]

De onderstaande vragen gaan over je persoonlijke en studiegerelateerde achtergrond.

1. Wat is je geslacht?
   - o Man
   - o Vrouw

2. Wat is je leeftijd?

   [ ] jaar

3. Wat is je geboorteland?

   [ ]

4. Wat is het geboorteland van je moeder?

   [ ]

5. Wat is het geboorteland van je vader?

   [ ]

6. Hoe is je woonsituatie?
   - o Ik woon in een studentenflat of studentenwoning (koop of huur)
   - o Ik woon in een eigen woning of appartement (koop of huur)
   - o Ik woon bij mijn ouders / verzorgers
   - o Anders, namelijk: [ ]

7. Aan welke universiteit studeer je?
   - o Vrije Universiteit van Amsterdam
   - o Universiteit van Amsterdam
   - o Technische Universiteit van Delft
   - o Wageningen UR
   - o Rijksuniversiteit Groningen

- Universiteit van Maastricht
- Universiteit van Leiden
- Technische Universiteit Eindhoven
- Radboud Universiteit Nijmegen
- Erasmus Universiteit
- Universiteit van Tilburg
- Universiteit van Utrecht
- Universiteit van Twente
- Anders, namelijk:

8. Welke opleiding volg je?

9. Kun je jouw opleiding typeren?
   - Bachelor programma
   - Master programma
   - Premaster / schakel programma
   - Anders, namelijk:

10. Wanneer was je voor het eerst ingeschreven bij je huidige opleiding?

    Datum (dd/mm/yy) :

De onderstaande vragen gaan over de universiteit waaraan je momenteel studeert en zoals deze er op dit moment uitziet.

11. Hoeveel uur per week besteed je gewoonlijk in en rondom de universiteit?

[ ] uur per week

12. In welk universiteitsgebouw volg je het vaakst onderwijs? (Met onderwijs bedoelen we hoorcolleges, werkgroepen en practica)

[ ]

De vragen 13 tot en met 18 gaan over het universiteitsgebouw aangegeven in vraag 12.

13. Wat vind je van de **buitenruimte** van het universiteitsgebouw waar je het vaakst onderwijs volgt? (Met buitenruimte bedoelen we de omgeving rondom het universiteitsgebouw, niet verder dan 5 minuten lopen vanaf de ingang)

    Vink bij elk woord het rondje aan dat het beste weergeeft wat je vindt van de buitenruimte.

	Niet	Een beetje	Nogal	Heel erg
Vrolijk	O	O	O	O
Somber	O	O	O	O
Lelijk	O	O	O	O
Rustgevend	O	O	O	O
Inspirerend	O	O	O	O
Hectisch	O	O	O	O
Aantrekkelijk	O	O	O	O
Saai	O	O	O	O
Smakeloos	O	O	O	O
Natuurrijk	O	O	O	O

14. Hoe tevreden ben je met de **buitenruimte** van het universiteitsgebouw waar je het vaakst onderwijs volgt?

    Druk je tevredenheid uit in een rapportcijfer van 1 tot 10 (1= zeer ontevreden, 10=zeer tevreden).

1	2	3	4	5	6	7	8	9	10
O	O	O	O	O	O	O	O	O	O

15. Wat vind je van de **collegezalen** van het universiteitsgebouw waar je het vaakst onderwijs volgt?

Vink bij elk woord het rondje aan dat het beste weergeeft wat je vindt van de collegezalen.

	Niet	Een beetje	Nogal	Heel erg
Vrolijk	O	O	O	O
Somber	O	O	O	O
Lelijk	O	O	O	O
Rustgevend	O	O	O	O
Inspirerend	O	O	O	O
Hectisch	O	O	O	O
Aantrekkelijk	O	O	O	O
Saai	O	O	O	O
Smakeloos	O	O	O	O
Natuurrijk	O	O	O	O

16. Hoe tevreden ben je met de **collegezalen** van het universiteitsgebouw waar je het vaakst onderwijs volgt?

Druk je tevredenheid uit in een rapportcijfer van 1 tot 10 (1= zeer ontevreden, 10=zeer tevreden).

1	2	3	4	5	6	7	8	9	10
O	O	O	O	O	O	O	O	O	O

17. Wat vind je van de **werkgroepruimtes** van het universiteitsgebouw waar je het vaakst onderwijs volgt?

Vink bij elk woord het rondje aan dat het beste weergeeft wat je vindt.

	Niet	Een beetje	Nogal	Heel erg
Vrolijk	O	O	O	O
Somber	O	O	O	O
Lelijk	O	O	O	O
Rustgevend	O	O	O	O
Inspirerend	O	O	O	O
Hectisch	O	O	O	O
Aantrekkelijk	O	O	O	O

Saai	O	O	O	O
Smakeloos	O	O	O	O
Natuurrijk	O	O	O	O

18. Hoe tevreden ben je met de **werkgroepruimtes** van het universiteitsgebouw waar je het vaakst onderwijs volgt? Druk je tevredenheid uit in een rapportcijfer van 1 tot 10 (1= zeer ontevreden, 10=zeer tevreden).

1	2	3	4	5	6	7	8	9	10
O	O	O	O	O	O	O	O	O	O

19. Welk universiteitsgebouw gebruik je het vaakst voor zelfstudie?

20. Wat voor soort ruimte, in het universiteitsgebouw aangegeven in vraag 19, gebruik je het vaakst voor zelfstudie?
    - o De universiteitsbibliotheek
    - o Een stilteruimte
    - o Een studieruimte
    - o Een computerruimte
    - o Anders, namelijk:

21. Wat vind je van de **studieruimte** die je het vaakst gebruikt (de ruimte die je hebt aangegeven in vraag 20)? Vink bij elk woord het rondje aan dat het beste weergeeft wat je vindt van de studieruimte

	Niet	Een beetje	Nogal	Heel erg
Vrolijk	O	O	O	O
Somber	O	O	O	O
Lelijk	O	O	O	O
Rustgevend	O	O	O	O
Inspirerend	O	O	O	O
Hectisch	O	O	O	O
Aantrekkelijk	O	O	O	O

Saai	O	O	O	O
Smakeloos	O	O	O	O
Natuurrijk	O	O	O	O

22. Hoe tevreden ben je met de **studieruimte** die je het vaakst gebruikt (de ruimte die je hebt aangegeven in vraag 20).

    Druk je tevredenheid uit in een rapportcijfer van 1 tot 10 (1= zeer ontevreden, 10=zeer tevreden).

1	2	3	4	5	6	7	8	9	10
O	O	O	O	O	O	O	O	O	O

De volgende vragen gaan over de omgeving van het universiteitsgebouw waar je het vaakst onderwijs volgt (het gebouw dat je hebt aangegeven in vraag 12). De eerste vier vragen gaan over de hoeveelheid en de kwaliteit van het groen in en rondom jouw universiteit. Met groen bedoelen we natuur of natuurlijke elementen zoals bomen, planten en grasvelden. De vragen daarna gaan over de voorzieningen in en rondom je universiteit.

23. Hoe tevreden ben je met de **hoeveelheid groen in het universiteitsgebouw** waar je het vaakst onderwijs volgt?

    Druk je tevredenheid uit in een rapportcijfer van 1 tot 10 (1= zeer ontevreden, 10=zeer tevreden).

1	2	3	4	5	6	7	8	9	10
O	O	O	O	O	O	O	O	O	O

24. Hoe tevreden ben jij met de **kwaliteit van het groen in het universiteitsgebouw** waar je het vaakst onderwijs volgt?

    Druk je tevredenheid uit in een rapportcijfer van 1 tot 10 (1= zeer ontevreden, 10=zeer tevreden).

1	2	3	4	5	6	7	8	9	10
O	O	O	O	O	O	O	O	O	O

25. Hoe tevreden ben je met de **hoeveelheid groen** in de **buitenruimte** van het universiteitsgebouw waar je het vaakst onderwijs volgt? (Met buitenruimte bedoelen we de omgeving rondom het universiteitsgebouw, niet verder dan 5 minuten lopen vanaf de ingang)

    Druk je tevredenheid uit in een rapportcijfer van 1 tot 10 (1= zeer ontevreden, 10=zeer tevreden).

1	2	3	4	5	6	7	8	9	10

O	O	O	O	O	O	O	O	O	O

26. Hoe tevreden ben je met **de kwaliteit van het groen** in de **buitenruimte** van het universiteitsgebouw waar je het vaakst onderwijs volgt? (Met buitenruimte bedoelen we de omgeving rondom het universiteitsgebouw, niet verder dan 5 minuten lopen vanaf de ingang)

    Druk je tevredenheid uit in een rapportcijfer van 1 tot 10 (1= zeer ontevreden, 10=zeer tevreden).

1	2	3	4	5	6	7	8	9	10
O	O	O	O	O	O	O	O	O	O

27. Hoe vaak koop je gewoonlijk eten of drinken op de universiteit?
    - (Bijna) nooit [Ga door naar vraag 29]
    - 1 x per maand
    - 2 – 3 x per maand
    - 1 – 4 x week
    - (Bijna) dagelijks

28. Wanneer je eten of drinken koopt op de universiteit, waar koop je dit dan?
    - In de universiteitskantine of in het universiteitsrestaurant
    - Bij de foodcorners of koffiecorners op de universiteit
    - Uit de automaat op de universiteit
    - Bij de universiteitssupermarkt
    - Anders, namelijk:

29. Hieronder staan stellingen over bepaalde voorzieningen in en rondom de universiteit. In welke mate ben je het eens met deze stellingen en hoe belangrijk vind je dit?

	Vink bij elke stelling het rondje aan dat het beste weergeeft wat je vindt.					Vink bij iedere stelling het rondje aan dat het beste weergeeft hoe belangrijk je dit vindt.				
	Zeer oneens	On-eens	Neutraal	Eens	Zeer eens	Zeer on-belangrijk	On-belangrijk	Neutraal	Belangrijk	Zeer belangrijk
Er is genoeg gezonde voeding verkrijgbaar in en rondom mijn universiteit	O	O	O	O	O	O	O	O	O	O
Er is genoeg groente en fruit verkrijgbaar in en rondom mijn universiteit	O	O	O	O	O	O	O	O	O	O

Er zijn veel mogelijkheden om snacks, snoep en frisdrank te kopen in en rondom mijn universiteit	O	O	O	O	O	O	O	O	O	O
Er zijn genoeg mogelijkheden om lichamelijk actief te zijn in en rondom mijn universiteit	O	O	O	O	O	O	O	O	O	O
Er zijn genoeg mogelijkheden om te ontspannen in en rondom mijn universiteit	O	O	O	O	O	O	O	O	O	O

30. Hoe belangrijk vind je het dat de universiteit de onderstaande voorzieningen aanbiedt en zou je meer groente en fruit eten als deze voorzieningen worden aangeboden?

	Vink bij iedere voorziening het rondje aan wat het beste weergeeft hoe belangrijk je dit vindt.					Zou je meer groente en fruit eten als je dit wordt aangeboden	
	Zeer onbelangrijk	Onbelangrijk	Neutraal	Belangrijk	Zeer belangrijk	Nee	Ja
Universiteitssupermarkt met betaalbare groente en fruit	O	O	O	O	O	O	O
Universiteitskantine met betaalbare groente en fruit	O	O	O	O	O	O	O
Universiteitsmoestuin / groentetuin	O	O	O	O	O	O	O
Wekelijkse lokale groente-en / of fruitmarkt in het universiteitsgebouw of in de directe omgeving	O	O	O	O	O	O	O
Groente- en/of fruitpakketten om af te halen op de universiteit	O	O	O	O	O	O	O

In de volgende vragen krijg je foto's te zien van verschillende universiteitsruimtes. Belangrijk hierbij is dat je NIET de kwaliteit van de foto beoordeeld maar WEL de ruimte en de setting afgebeeld op de foto

31. Wat vind je van de ruimte op de foto?

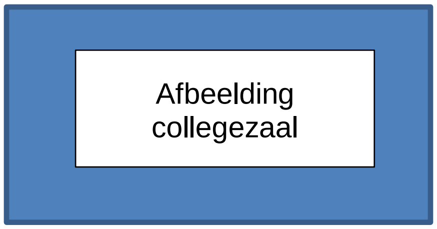

Vink bij elke stelling het rondje aan welke het beste weergeeft wat je vindt van de setting afgebeeld op de foto.

	Zeer oneens	Oneens	Neutraal	Eens	Zeer eens
De ruimte is aangenaam	O	O	O	O	O
De ruimte is aantrekkelijk	O	O	O	O	O
Ik zou graag les krijgen in de ruimte	O	O	O	O	O
De ruimte stimuleert om mijn aandacht bij de studiestof te houden	O	O	O	O	O
De ruimte stimuleert tot creatief nadenken	O	O	O	O	O

32. Wat vind je van de ruimte op de foto?

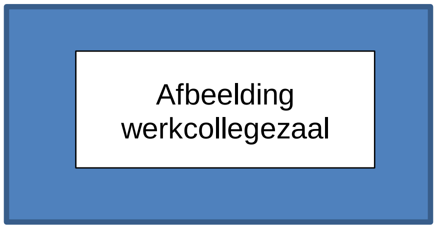

Vink bij elke stelling het rondje aan welke het beste weergeeft wat je vindt van de setting afgebeeld op de foto.

	Zeer oneens	Oneens	Neutraal	Eens	Zeer eens
De ruimte is aangenaam	O	O	O	O	O
De ruimte is aantrekkelijk	O	O	O	O	O
Ik zou graag les krijgen in de ruimte	O	O	O	O	O
De ruimte stimuleert om mijn aandacht bij de studiestof te houden	O	O	O	O	O
De ruimte stimuleert tot creatief nadenken	O	O	O	O	O

33. Wat vind je van de ruimte op de foto?

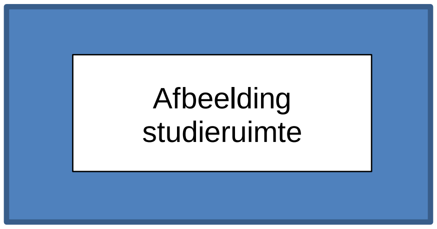

Vink bij elke stelling het rondje aan welke het beste weergeeft wat je vindt van de setting afgebeeld op de foto.

	Zeer oneens	Oneens	Neutraal	Eens	Zeer eens
De ruimte is aangenaam	O	O	O	O	O
De ruimte is aantrekkelijk	O	O	O	O	O
Ik zou graag studeren in de ruimte	O	O	O	O	O
De ruimte stimuleert om mijn aandacht bij de studiestof te houden	O	O	O	O	O
De ruimte stimuleert tot creatief nadenken	O	O	O	O	O

34. Wat vind je van de ruimte op de foto?

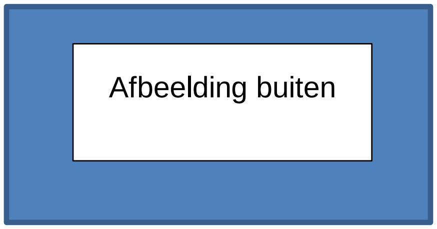

Vink bij elke stelling het rondje aan welke het beste weergeeft wat je vindt van de setting afgebeeld op de foto.

	Zeer oneens	Oneens	Neutraal	Eens	Zeer eens
De ruimte is aangenaam	O	O	O	O	O
De ruimte is aantrekkelijk	O	O	O	O	O
Ik zou graag verblijven in de ruimte	O	O	O	O	O
Als ik 20 minuten in deze ruimte zou verblijven dan kom ik tot rust	O	O	O	O	O
Als ik 20 minuten in deze ruimte zou verblijven dan heb ik vernieuwde energie	O	O	O	O	O
Als ik aan het eind van een week vol examens en intens studeren er doorheen zit, geestelijk vermoeid ben en me niet meer kan concentreren dan zou ik in deze ruimte mijn concentratie kunnen terugvinden.	O	O	O	O	O
Ik zou de ruimte gebruiken voor sociale doeleinde	O	O	O	O	O
Ik zou de ruimte gebruiken voor lichamelijke beweging	O	O	O	O	O

35. Bekijk de vier foto's. In welke van de vier ruimtes zou je het liefst onderwijs volgen

- Foto 1
- Foto 2
- Foto 3
- Foto 4

36. Waarom zou je het liefst onderwijs volgen in de ruimte aangegeven in vraag 35?

- Deze ruimte is het meest aantrekkelijk
- Deze ruimte is het meest inspirerend
- Deze ruimte is het meest rustgevend
- Deze ruimte is het meest vrolijk
- Anders, namelijk:

De volgende vragen gaan over je leefstijl zoals groente en fruit inname, bewegen, roken, alcohol en slaap. Daarnaast stellen we enkele vragen over een groene omgeving.

37. Hoeveel dagen per week eet je gewoonlijk fruit?

Minder dan 1	1	2	3	4	5	6	7 dagen per week
O	O	O	O	O	O	O	O

38. Op de dagen dat je fruit eet, hoeveel porties eet je dan gewoonlijk?

(1 portie fruit is bijvoorbeeld een middelgrote appel of 2 mandarijntjes. Bij klein fruit, zoals kersen, kunt je een handje vol voor 1 portie tellen)

Minder dan 1	1	2	3	4	5	Meer dan 5 porties
O	O	O	O	O	O	O

39. Hoeveel dagen per week eet je gewoonlijk gekookte of gebakken groente, salade of rauwkost? (Groenten in eenpansgerechten, zoals stamppotten, tellen mee, een blaadje sla op bijvoorbeeld een broodje gezond telt niet mee)

Minder dan 1	1	2	3	4	5	6	7 dagen per week
O	O	O	O	O	O	O	O

40. Op de dagen dat je gekookte of gebakken groente, salade of rauwkost eet, hoeveel opscheplepels eet je dan gewoonlijk? (Een opscheplepel is ongeveer 50 gram)

Minder dan 1	1	2	3	4	5	Meer dan 5 opscheplepels
O	O	O	O	O	O	O

41.

42. Hoeveel dagen per week beweeg je tenminste 30 minuten op een matig intensief niveau zoals wandelen en fietsen?

Minder dan 1	1	2	3	4	5	6	7 dagen per week
O	O	O	O	O	O	O	O

43. Hoeveel dagen per week beweeg je tenminste 20 minuten op een zwaar intensief niveau zoals fitness, voetbal of tennis ?

Minder dan 1	1	2	3	4	5	6	7 dagen per week
O	O	O	O	O	O	O	O

44. Rook je weleens?
- Nee [Ga verder met vraag 45]
- Ja

45. Rook je elke dag van de week?
- Nee
- Ja: Hoeveel eenheden rook je dan gewoonlijk?

[ ] eenheden

46. Hoeveel dagen per week drink je alcohol?

Ik drink nooit alcohol	1	2	3	4	5	6	7 dagen per week
O	O	O	O	O	O	O	O

47. Op de dagen dat je alcohol drink, hoeveel glazen alcohol drink je dan?

Minder dan 1	1	2	3	4	5	6 glazen of meer
O	O	O	O	O	O	O

48. Hoe zou je de hoeveelheid stress die je ervaart beoordelen?

Geen stress				Extreem veel stress
O	O	O	O	O

49. Hoe goed kun je omgaan met stress?

Heel goed:<br>ik kan stress gemakkelijk van mij afschudden				Zeer slecht:<br>stress vreet mij op
O	O	O	O	O

50. Hoe laat ga je ’s avonds gewoonlijk slapen? (Geef het tijdstip aan in cijfers, zoals 22.30)

Door de weeks:	
In het weekend:	

51. Hoe laat wordt je ’s ochtends gewoonlijk wakker? (Geef het tijdstip aan in cijfers, zoals 07.00)

Door de weeks:	
In het weekend:	

51. Hoe lang ben je? (Het gaat hier om lengte in centimeters, zonder schoenen)

	Centimeter

52. Hoeveel kilo weeg je? (Het gaat om het gewicht in hele kilo’s, in de ochtend gewogen, zonder kleren)

	Kilo

53. Hoe vaak zoek je gewoonlijk doelbewust een groene omgeving op zoals een bos, stadspark of botanische tuin?

- (Bijna) nooit
- 1 x per maand

- 2 – 3 x per maand
- 1 - 4 x per week
- (Bijna) dagelijks

54. Wanneer je doelbewust een groene omgeving bezoekt, hoe lang duurt je bezoek dan gewoonlijk?

[ ] Minuten

55. In hoeverre ben je een natuurliefhebber? Druk dit uit in een rapportcijfer van 1 tot 10.

1	2	3	4	5	6	7	8	9	10
O	O	O	O	O	O	O	O	O	O

Bedankt voor je deelname !

The following questions concern your personal and academic-related background.

1. What is your gender?
   - o Male
   - o Female

2. What is your age?

   [ ] Years

3. What is your country of birth?

   [ ]

4. What is your mother's country of birth?

   [ ]

5. What is your father's country of birth?

   [ ]

6. What is your living situation?
   - o I live in shared student housing
   - o I live in my own house or apartment (owner or rent)
   - o I live with my parents / caretakers
   - o Other, being: [ ]

7. At which university do you study?
   - o VU University Amsterdam
   - o University of Amsterdam
   - o Delft University of Technology
   - o Wageningen UR
   - o University of Groningen
   - o Maastricht University
   - o Leiden University
   - o Eindhoven University of Technology
   - o Radboud University Nijmegen
   - o Erasmus University
   - o Tilburg University
   - o Utrecht University
   - o University of Twente
   - o Other, being: [ ]

8. What is your course of study?

   [ ]

9. Please specify your study type?
   - o Bachelor
   - o Masters
   - o Premaster / transition year
   - o Other, being: [ ]

10. When where you first registered in your current study?

    [ ] Date (dd/mm/yy)

The following questions are about the university where you currently study related to the present period.

11. How many hours per week do you usually spend in and around the university?

    [ ] Hours a week

12. Which university building do you use for education most often? (Education refers to lectures, workgroups and practice lessons)

    [ ]

The questions 13 through 18 concerns the university building indicated in question 12.

13. What do you think of the **outdoor space** of the university building that you use for education most often? (The outdoor space refers to the environment around the university building within a five minute walk)

    For each word, please check the box that best represents your assessment.

	Not	A little	Quite	Very
Cheerful	O	O	O	O
Gloomy	O	O	O	O
Ugly	O	O	O	O
Calming	O	O	O	O
Inspiring	O	O	O	O
Hectic	O	O	O	O
Attractive	O	O	O	O
Drab or dull	O	O	O	O
Tacky	O	O	O	O
Natural	O	O	O	O

14. How satisfied are you with the **outdoor space** of the university building that you use for education most often?

    Express your satisfaction on a scale from 1 to 10 (1=very unsatisfied, 10= very satisfied).

1	2	3	4	5	6	7	8	9	10
O	O	O	O	O	O	O	O	O	O

15. What do you think of the **lecture halls** of the university building that you use for education most often?

    For each word, please check the box that best represents your assessment.

	Not	A little	Quite	Very
Cheerful	O	O	O	O
Gloomy	O	O	O	O
Ugly	O	O	O	O
Calming	O	O	O	O
Inspiring	O	O	O	O
Hectic	O	O	O	O
Attractive	O	O	O	O
Drab or dull	O	O	O	O
Tacky	O	O	O	O
Natural	O	O	O	O

16. How satisfied are you with the **lecture halls** of the university building that you use for education most often?

Express your satisfaction on a scale from 1 to 10 (1=very unsatisfied, 10= very satisfied).

1	2	3	4	5	6	7	8	9	10
O	O	O	O	O	O	O	O	O	O

17. What do you think of the **workgroup rooms** of the university building that you use for education most often?

For each word, please check the box that best represents your assessment.

	Not	A little	Quite	Very
Cheerful	O	O	O	O
Gloomy	O	O	O	O
Ugly	O	O	O	O
Calming	O	O	O	O
Inspiring	O	O	O	O
Hectic	O	O	O	O
Attractive	O	O	O	O
Drab or dull	O	O	O	O
Tacky	O	O	O	O
Natural	O	O	O	O

18. How satisfied are you with the **workgroup rooms** of the university building that you use for education most often?

Express your satisfaction on a scale from 1 to 10 (1=very unsatisfied, 10= very satisfied).

1	2	3	4	5	6	7	8	9	10
O	O	O	O	O	O	O	O	O	O

19. Which university building do you most often use for learning and studying?

20. What kind of area in the building, indicated in question 19, do you most often use for learning and studying?
- o The university library
- o A silent study room
- o A study room
- o A computer area
- o Other, being:

21. What do you think of the **area** that you most often use **for learning and studying** (the area indicated in question 20)?

For each word, please check the box that best represents your assessment.

	Not	A little	Quite	Very
Cheerful	O	O	O	O
Gloomy	O	O	O	O
Ugly	O	O	O	O
Calming	O	O	O	O
Inspiring	O	O	O	O
Hectic	O	O	O	O
Attractive	O	O	O	O
Drab or dull	O	O	O	O
Tacky	O	O	O	O
Natural	O	O	O	O

22. How satisfied are you with the **area** that you most often use **for learning and studying** (the area that you indicated in question 20)?

Express your satisfaction on a scale from 1 to 10 (1=very unsatisfied, 10= very satisfied).

1	2	3	4	5	6	7	8	9	10
O	O	O	O	O	O	O	O	O	O

The following questions concern the environment of the university building that you most often use for education (the building indicated in question 12). The first four questions are about the quantity and quality of the greenery in the university environment. Greenery refers to nature or nature elements such as trees, plants or lawns. The subsequent questions are about the facilities in the university environment.

23. How satisfied are you with the **quantity of greenery inside** the university building that you use for education most often?

Express your satisfaction on a scale from 1 to 10 (1=very unsatisfied, 10= very satisfied).

1	2	3	4	5	6	7	8	9	10
O	O	O	O	O	O	O	O	O	O

24. How satisfied are you with the **quality of greenery inside** the university building that you use for education most often?

Express your satisfaction on a scale from 1 to 10 (1=very unsatisfied, 10= very satisfied).

1	2	3	4	5	6	7	8	9	10
O	O	O	O	O	O	O	O	O	O

25. How satisfied are you with the **quantity of greenery in the outside space of the university building** that you use for education most often? (The outdoor space refers to the environment around the university building within a five minute walk)

Express your satisfaction on a scale from 1 to 10 (1=very unsatisfied, 10= very satisfied).

1	2	3	4	5	6	7	8	9	10
O	O	O	O	O	O	O	O	O	O

26. How satisfied are you with the **quality of greenery in the outside space of the university building** that you use for education most often? (The outdoor space refers to the environment around the university building within a five minute walk)

Express your satisfaction on a scale from 1 to 10 (1=very unsatisfied, 10= very satisfied).

1	2	3	4	5	6	7	8	9	10
O	O	O	O	O	O	O	O	O	O

27. How often do you usually buy food or drinks at the university?
    - (Almost) never (skip to 29)
    - Once per month
    - 2 to 3 times per month
    - 1 to 4 times per week
    - (Almost) daily

28. If you buy food or drinks at the university where do you usually buy it?
    - At the university cafeteria or university restaurant
    - At the food corners or coffee corners at the university
    - Out of a vending machine at the university
    - At the university supermarket
    - Other, being:

29. Below statements on certain facilities in and around the university are given. To what extent do you agree with these statements and how important are these facilities?

	Please check the box that best represents your assessment					Please check the box that best represents how important you think this facility is				
	Strongly disagree	Disagree	Neutral	Agree	Strongly agree	Very Un-important	Un-important	Neutral	Important	Very important
There is plenty of healthy foods available in and around my university	O	O	O	O	O	O	O	O	O	O
There are plenty fruits and vegetables available in and around my university	O	O	O	O	O	O	O	O	O	O
There are many opportunities to purchase snacks, candy or sodas in and around my university	O	O	O	O	O	O	O	O	O	O
There are plenty opportunities to be physically active in and around my university	O	O	O	O	O	O	O	O	O	O
There are plenty opportunities to relax in and around my university	O	O	O	O	O	O	O	O	O	O

30. How important is it to you that the university offer the following facilities and, if so, would you eat more fruits and vegetables with access to these facilities?

	Please check the box that best represent how important you think this facility is					Would you eat more fruits and vegetables?	
	Very unimportant	Unimportant	Neutral	Important	Very important	No	Yes
University supermarket with affordable fruits and vegetables	O	O	O	O	O	O	O
University canteen with affordable fruits and vegetables	O	O	O	O	O	O	O
A university vegetable garden	O	O	O	O	O	O	O
Weekly local farmers market in the university environment	O	O	O	O	O	O	O
Readymade vegetable packages that can be picked up at the university	O	O	O	O	O	O	O

The following questions are about your assessment of photos of four university spaces. You are not asked to assess the quality of the photo, but the setting depicted in the photo.

31. What do you think of the setting depicted in the photo? For each statement, please check the box that best represents your assessment of the setting.

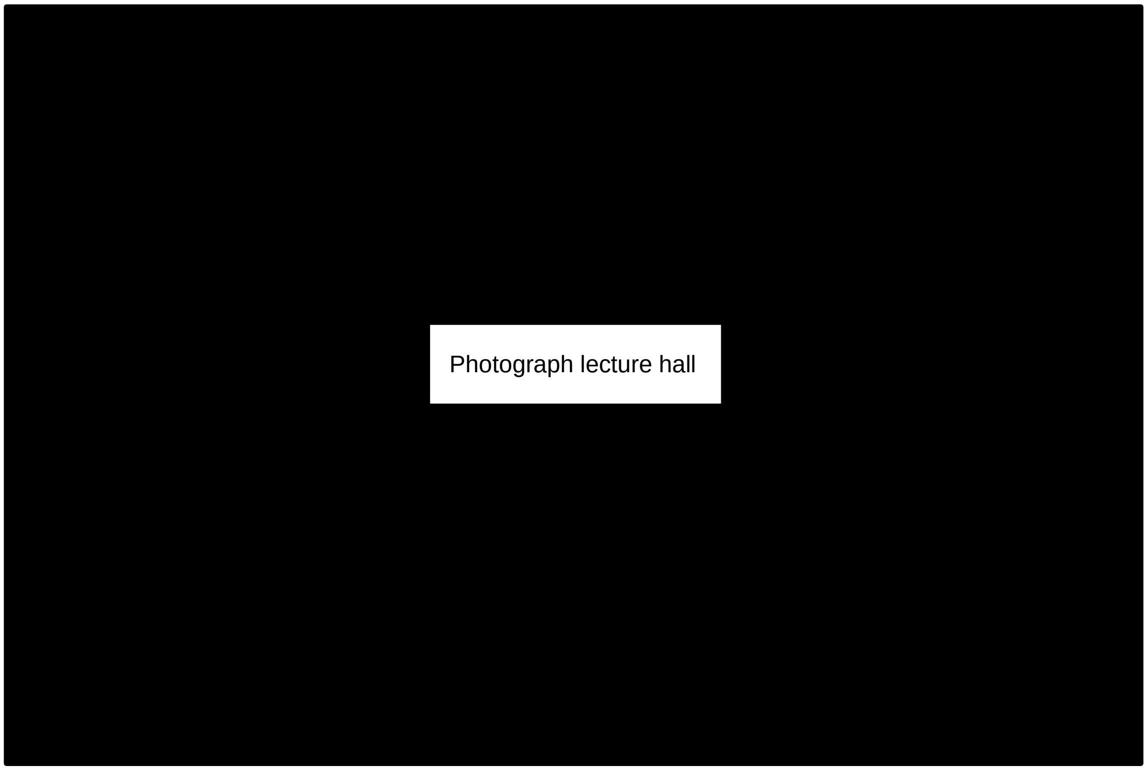

	Strongly disagree	Disagree	Neutral	Agree	Strongly agree
The setting is pleasant	O	O	O	O	O
The setting is attractive	O	O	O	O	O
I would like to be educated in this setting	O	O	O	O	O
This setting stimulates me to keep my focus on my study materials	O	O	O	O	O
This setting stimulates my creative thinking	O	O	O	O	O

32. What do you think of the setting depicted in the photo?

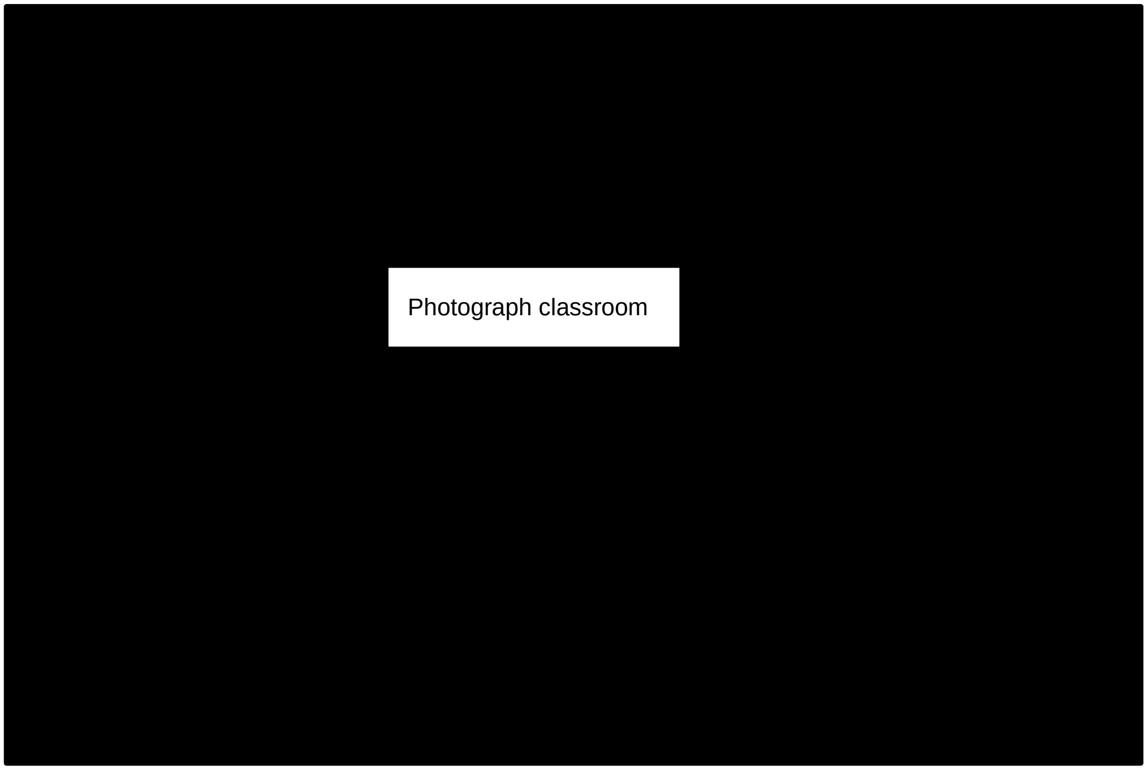

For each statement, please check the box that best represents your assessment of the setting.

	Strongly disagree	Disagree	Neutral	Agree	Strongly agree
The setting is pleasant	O	O	O	O	O
The setting is attractive	O	O	O	O	O
I would like to be educated in this setting	O	O	O	O	O
This setting stimulates me to keep my focus on my study materials	O	O	O	O	O
This setting stimulates my creative thinking	O	O	O	O	O

33. What do you think of the setting depicted in the photo?

34.

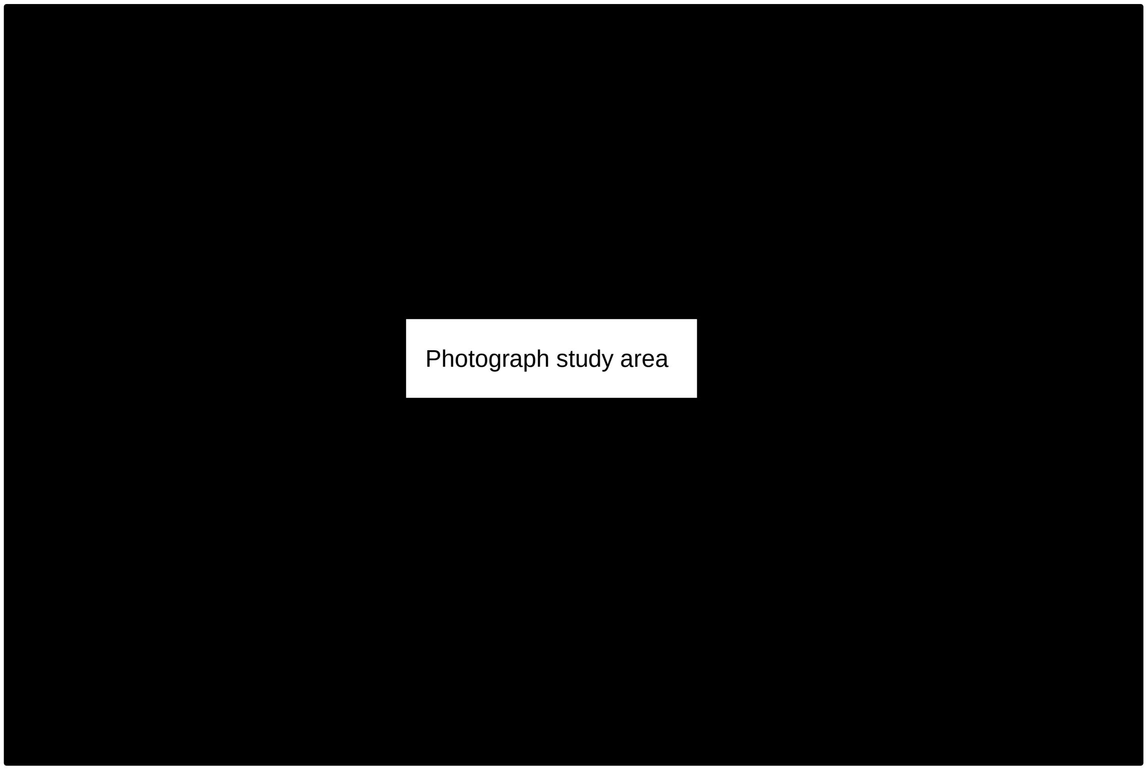

For each statement, please check the box that best represents your assessment of the setting.

	Strongly disagree	Disagree	Neutral	Agree	Strongly agree
The setting is pleasant	O	O	O	O	O
The setting is attractive	O	O	O	O	O
I would like to study in this setting	O	O	O	O	O
This setting stimulates me to keep my focus on my study materials	O	O	O	O	O
This setting stimulates my creative thinking	O	O	O	O	O

35. What do you think of the setting depicted in the photo?

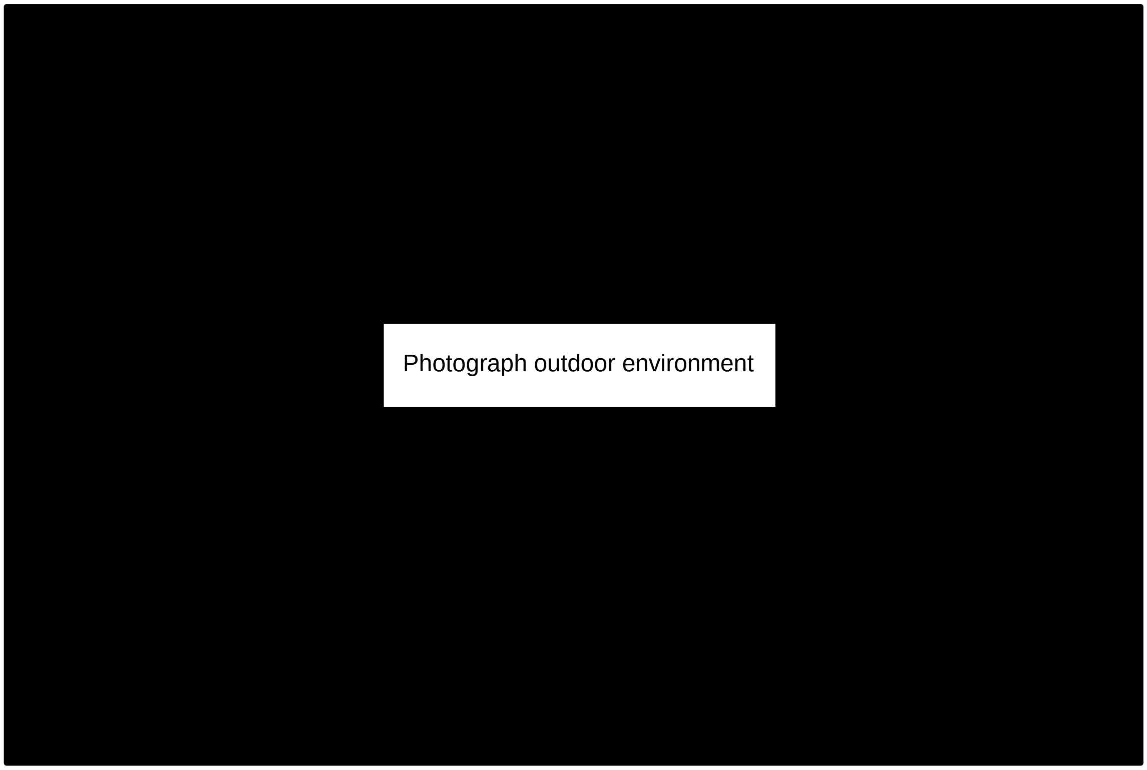

For each statement, please check the box that best represents your assessment of the setting.

	Strongly disagree	Disagree	Neutral	Agree	Strongly agree
The setting is pleasant	O	O	O	O	O
The setting is attractive	O	O	O	O	O
I would like stay in this setting	O	O	O	O	O
If I would stay in this setting for 20 minutes I would feel that I had come to rest	O	O	O	O	O
If I would stay in this setting for 20 minutes that I would feel that I have renewed energy	O	O	O	O	O
If, at the end of a week of exams and intense study I am mentally exhausted and unable to concentrate than I could regain my concentration in this setting	O	O	O	O	O
I would use this setting for socializing	O	O	O	O	O
I would use this setting for physical activity	O	O	O	O	O

36. Assess the four photos. In which setting would you prefer to be educated ?

O Photo 1 O Photo 2

O Photo 3 O Photo 4

37. Why do you prefer the setting that you indicated in question 35
   - This is the most attractive setting
   - This is the most inspiring setting
   - This is the most peaceful setting
   - This is the most cheerful setting
   - Other, being:

The following questions are about your lifestyle such as fruit and vegetable intake, exercise, smoking, alcohol and sleep. Thereafter there are three questions concerning greenery.

38. How many days per week do you usually eat fruit?

Less than 1	1	2	3	4	5	6	7 days per week
O	O	O	O	O	O	O	O

39. On the days that you eat fruit, how many portions do you usually take? (1 portion is, for example, a medium sized apple or 2 mandarins. With small fruits such as cherries or grapes, 1 hand-full equals 1 portion)

Less than 1	1	2	3	4	5	More than 5 portions
O	O	O	O	O	O	O

40. How many days per week do you usually eat vegetables? (Examples are vegetable side-dishes or salads. Vegetables that are part of a large dish that also contains non-vegetable ingredients also counts. Lettuce on a sandwich, however, does not)

Less than 1	1	2	3	4	5	6	7 days per week
O	O	O	O	O	O	O	O

41. On the days that you eat vegetables how many servings do you usually eat? (One servings equals a proximally 50 grams).

Less than 1	1	2	3	4	5	More than 5 servings
O	O	O	O	O	O	O

42. How many days per week are you physically active for at least 30 minutes on a moderate level such as walking and cycling?

Less than 1	1	2	3	4	5	6	7 days per week
O	O	O	O	O	O	O	O

43. How many days per week are you physically active for at least 20 minutes on a heavily intense levels such as soccer, tennis or at the gym?

Less than 1	1	2	3	4	5	6	7 days per week
O	O	O	O	O	O	O	O

44. Do you ever smoke?
- ○ No [Skip to 45]
- ○ Yes

45. Do you smoke every day?
- ○ No
- ○ Yes: How many units do you smoke on an average day

Units

46. How many days per week do you usually drink alcoholic beverages?

I never drink alcohol [skip to 47]	1	2	3	4	5	6	7 days per week
O	O	O	O	O	O	O	O

47. On the days that you drink alcoholic beverages, how many glasses do you drink?

Less than 1	1	2	3	4	5	6 glasses or more
O	O	O	O	O	O	O

48. How would you rate the level of stress that you usually experience?

No stress				Extreme stress
O	O	O	O	O

49. How well can you cope with stress?

Excellently: I can shake of stress easily				Very poorly: stress eats me away
O	O	O	O	O

50. At what time do you usually go to sleep at night?

During the week	
In the weekend:	

51. At what time do you usually wake up in the morning?

During the week	
In the weekend:	

52. How tall are you? (Please state your length in centimetres without shoes)

[ ] Centrimeters

53. What is your weight? (Please state your weight in whole kilos without clothes)

[ ] Kilos

54. How often do you intentionally visit green environments? *(green environments refers to natural environments such as forests, parks or botanic gardens)*
   - ○ (Almost) Never [Skip to 55]
   - ○ 1 time per month
   - ○ 2 -3 times per month
   - ○ 1 -4 times per week
   - ○ (Almost) Daily

55. When you intentionally visit a green environment, how long do you usually stay?

[ ] *minutes*

56. To what extend are you a nature lover?

Rate this on a scale from 1 to 10 (1 =you dislike nature, 10= you love nature)

1	2	3	4	5	6	7	8	9	10
O	O	O	O	O	O	O	O	O	O
